# Exploring the benefits of participation in community-based running and walking events: a cross-sectional survey of *parkrun* participants

**DOI:** 10.1186/s12889-021-11986-0

**Published:** 2021-11-02

**Authors:** Helen Quirk, Alice Bullas, Steve Haake, Elizabeth Goyder, Mike Graney, Chrissie Wellington, Robert Copeland, Lindsey Reece, Clare Stevinson

**Affiliations:** 1 School of Health and Related Research, The University of Sheffield, 30 Regent St, Sheffield, S1 4DA UK; 2 Advanced Wellbeing Research Centre, Sheffield Hallam University, Sheffield, UK; 3 parkrun UK, Middlesex, UK; 4 Prevention Research Collaboration, School of Public Health, University of Sydney, Sydney, New South Wales Australia; 5 School of Sport, Exercise and Health Sciences, Loughborough University, Loughborough, UK

**Keywords:** Cross-sectional study, Inequalities, Deprivation, Physical activity, Community event

## Abstract

**Background:**

Whilst the benefits of physical activity for health and wellbeing are recognised, population levels of activity remain low. Significant inequalities exist, with socioeconomically disadvantaged populations being less physically active and less likely to participate in community events. We investigated the perceived benefits from participation in a weekly running/walking event called *parkrun* by those living in the most socioeconomically deprived areas and doing the least physical activity.

**Methods:**

A cross-sectional online survey was emailed to 2,318,135 *parkrun* participants in the UK. Demographic and self-reported data was collected on life satisfaction, happiness, health status, physical activity, motives, and the perceived benefits of *parkrun*. Motivation, health status and benefits were compared for sub-groups defined by physical activity level at *parkrun* registration and residential Index of Multiple Deprivation.

**Results:**

60,000 completed surveys were received (2.7% of those contacted). Respondents were more recently registered with *parkrun* (3.1 v. 3.5 years) than the *parkrun* population and had a higher frequency of *parkrun* participation (14.5 v. 3.7 *parkruns* per year). Those inactive at registration and from deprived areas reported lower happiness, lower life satisfaction and poorer health compared to the full sample. They were more likely to want to improve their physical health, rather than get fit or for competition. Of those reporting less than one bout of activity per week at registration, 88% (87% in the most deprived areas) increased their physical activity level and 52% (65% in the most deprived areas) reported improvements to overall health behaviours. When compared to the full sample, a greater proportion of previously inactive respondents from the most deprived areas reported improvements to fitness (92% v. 89%), physical health (90% v. 85%), happiness (84% v. 79%) and mental health (76% v. 69%).

**Conclusion:**

The least active respondents from the most socioeconomically deprived areas reported increases to their activity levels and benefits to health and wellbeing since participating in *parkrun*. Whilst the challenge of identifying *how* community initiatives like *parkrun* can better engage with underrepresented populations remains, if this can be achieved they could have a critical public health role in addressing inequalities in benefits associated with recreational physical activity.

**Supplementary Information:**

The online version contains supplementary material available at 10.1186/s12889-021-11986-0.

## Background

In its Global Action Plan on Physical Activity 2018–2030, the World Health Organization (WHO) identified a need for physical activity opportunities that use public spaces and engage whole communities [1]. Mass participation physical activity events have been recognised for their public health potential given their population reach, growing popularity and community context [2] and potential to engage patient populations [3]. However a criticism of mass sporting or physical activity events is that they can attract those who are already active and from more affluent areas [4, 5]. ‘One-off’ mass participation physical activity or sporting events may also have limited potential to leverage sustained behaviour change [2].

Starting in London, United Kingdom (UK) in 2004, *parkrun* is a charity that organises free, weekly (and thus regular), timed 5 km mass participation events for people to participate as runners/walkers (running or walking the 5 km course) or volunteers (permanent volunteers are responsible for the delivery of the event every week and episodic volunteers carry out event day volunteering duties such as marshalling, timekeeping, scanning barcodes, handing out finish tokens or tail walking). There is now a growing number of weekly *parkrun* events worldwide across 23 countries attracting millions of international participants and a global network of over 375,000 volunteers [6]. *parkrun (**www.parkrun.com**)* has been recognised in WHO’s Global Action Plan as a working example of “*regular mass-participation initiatives in public spaces, engaging whole communities, to provide free access to enjoyable and affordable, socially and culturally appropriate experiences of physical activity (page 66)*” [1].

*parkrun* events are organised by local volunteer teams and the opportunity to participate is open to all. Events are promoted as being inclusive to people from all backgrounds and abilities and research evidence would support its perceived inclusivity and ability to create a supportive environment [7–11]. Participation in *parkrun* is free: people register online and receive a unique ‘barcode’ containing their *parkrun* ID number that they take to any event across the world that is scanned and used to log attendance and completion time. An increasing proportion of events have been established in more socioeconomically disadvantaged areas in the UK, with higher population density resulting in better geographical access to events for those living in these areas [12, 13]. Inequalities in registration and participation persist despite *parkruns* being located closer to more socioeconomically disadvantaged areas [12, 14] with 13.1% of those participating at least once live in the most socioeconomically deprived areas of the UK (see Table 1).

**Table 1:** Population characteristics of parkrun participants

Parkrun participants (census date 3rd December 2018)		
n	1,549,806	
Proportion female	759,050	51.3%
Mean age of participants (years)	40.5	
Index of multiple deprivation		
n	1,385,961	
Quartile 1	181,561	13.1%
Physical activity level at registration		
n	1,656,006	
Less than one day per week of activity	109,296	6.6%
Mean years registered with parkrun	3.5	
Mean number of parkruns run/walked per year	3.7	

Previous research has demonstrated that individuals who live in socioeconomically deprived areas and are physically active may experience much better health and quality of life than their neighbours who are less active [15]. In a cohort study of 354 new *parkrun* participants in the UK, Stevinson and Hickson [16] also showed that *parkrun* participation is associated with significant positive changes in health and wellbeing over 6 and 12 months, including level of physical activity. However previous *parkrun* studies have not been designed to explore the relationship between socioeconomic deprivation and changes in physical activity for those inactive before participating and the perceived benefits of participation [16–18].

In 2018, a Health and Wellbeing Survey of UK *parkrun* participants was undertaken [19]. In this manuscript, we have used a large and diverse sample from that survey of *parkrun* runners/walkers and runners/walkers who volunteer to explore the following:

1. the motivation for first participating in *parkrun* as a runner or walker;
2. the self-reported health and wellbeing benefits from participation in *parkrun*.

We focus on sub-samples representing those who were previously inactive at registration, those from the most socioeconomically deprived areas, or both.

## Methods

### Procedure

Ethical approval for the study was granted by Sheffield Hallam University Research Ethics Committee on 24/07/2018 (reference number: ER7034346) and approval was granted from the *parkrun* Research Board. The study used an online survey, incorporating wherever possible existing measures used in health and wellbeing research. An advisory team, created using the *parkrun* Research Board and academics, were consulted to longlist and then shortlist the questions used in the survey. Each questionnaire or question was selected using the following criteria: relevance; validity; reliability; length; previous use. If suitable previous questionnaires or questions could not be identified, the research team developed study-specific questions to capture the outcome (as highlighted in the Methods section). The survey length and literacy were tested and re-tested via members of the research team and the advisory team. The reporting adheres to established standards for reporting internet-based surveys; The Checklist for Reporting Results of Internet E-Surveys (CHERRIES) [20].

### Population and participants

The sample was drawn from all *parkrun* registrants in the UK. Registrants received an email from *parkrun* containing a link to the survey. Survey participants had to be aged 16 or over and the survey was only available in online format and in the English language; there were no other explicit exclusion criteria. In this manuscript, we use the data from respondents who identified in the survey as runners/walkers and runners/walkers who also volunteer at *parkrun.* Runners/walkers are those who participate in *parkrun* by running or walking the 5 km course. Runners/walkers who also volunteer are those who participate in *parkrun* as a volunteer as well as a runner/walker. Findings relating to the health and wellbeing of *parkrun* volunteers and the perceived impact of volunteering at *parkrun* will be published separately.

### The survey

The measures in the survey are described fully in Additional file 1 with a full copy of the survey, including wording for consent. The list below describes the subset of measures used in this study.

#### Demographics

Demographic data included date of birth, gender, ethnicity, employment, home *parkrun* (the *parkrun* event they were most closely affiliated with), socioeconomic status and long-term health conditions.

Socioeconomic status was assessed using index of multiple deprivation (IMD) for Lower Level Super Output Areas (LSOA) derived from the postcode provided by the individual at *parkrun* registration. LSOAs are the smallest units from which Population Census data is compiled and onto which official data on socio-economic context is mapped by the Office of National Statistics [21]. IMD scores were classified into four quartiles Q1 to Q4 where Q1 represented the most deprived areas.

Long-term health conditions were recorded by self-report using the question: *“Are your day-to-day activities limited because of a health condition or disability which has lasted, or is expected to last, at least 12 months? Include conditions related to old age, sensory deficits, mobility problems, developmental conditions, learning impairments and mental health”* followed by a list of health conditions if they answered ‘yes, limited a lot’ or ‘yes, limited a little’ (56 health conditions were listed in total, plus an ‘other, please specify’ option). See Additional file 1 for the survey question.

One question asked participants to state whether they most closely identified as a *parkrun* runner/walker, a *parkrun* runner/walker *and* volunteer or a *parkrun* volunteer. Respondents were asked to provide their *parkrun* ID number to enable their survey responses to be matched to the *parkrun* database that holds their *parkrun* registration details (e.g. postcode, activity level at registration) and participation information (e.g. number of *parkruns* completed). See ‘*parkrun* data’ section below for more details.

#### Life satisfaction and happiness

Two of the four personal wellbeing questions asked in the UK’s Office of National Statistics (ONS) Annual Population Survey [22] were used as measures of life satisfaction and happiness: 1) *“Overall, how satisfied are you with your life nowadays?”* and 2) *“Overall, how happy did you feel yesterday?”* Statements were rated on a 10-point visual analogue scale where 0 is “not at all”, and 10 is “completely”. Life satisfaction and happiness were chosen from the four ONS measures because these aspects of wellbeing were not already captured in other measures used in the survey (see Additional file 1 for a full list of the questions used in the survey). Despite these ONS wellbeing measures being used extensively in large UK population surveys, there are no reported psychometric properties (e.g., validity). Each answer is taken at face value and cut-offs determine high and low scores.

#### Subjective health status

Subjective health status was measured using the EuroQoL visual analogue scale (EQ-VAS) [23] which asks: *“We would like to know how good or bad your health is TODAY. This scale is numbered from 0 to 100. 100 means the best health you can imagine. 0 means the worst health you can imagine. Please enter a number in the box below to indicate how your health is TODAY.”* The VAS was presented vertically with the label “the best/worst imaginable health” on the top/bottom and numbers ranging from 0 to 100 along the side. Permission was granted by EuroQol Research Foundation for its use. The construct validity of EQ-VAS has been reported as satisfactory [24].

#### Motivation for participating in *parkrun* as a runner/walker

Motivation for participation in *parkrun* was measured with a question developed by the research team for the purpose of this study: *“What motivated you to first participate at parkrun as a runner or walker?”* Respondents were asked to select a maximum of three answers out of a possible 21 motives. Examples of motives included; “*to improve my physical health*”, “*to improve my mental health*”, “*to manage my weight*”, “*to improve my happiness*”, “*to meet new people*” and “*to spend time with friends*” (see Additional file 1 for full list of motives). The 21 choices were displayed in randomised order to help reduce response bias. The final choice was “other” and, if selected, respondents were asked to specify the motive. Given that this was a study-specific question, there are no psychometric properties to report for this measure.

#### Self-reported physical activity

Self-reported physical activity was measured using three different measures: 1) a single item four-week recall physical activity question that is also asked at *parkrun* registration; 2) a single item 1 week recall physical activity question [25]; and 3) the International Physical Activity Questionnaire Short Form (IPAQ-SF) [26].

The four-week recall question asked: “*Over the last 4 weeks, how often have you done at least 30 minutes of moderate exercise (enough to raise your breathing rate)?”* Respondents could answer: less than once per week, about once per week, about twice per week, about three times per week, four or more times per week, rather not say, don’t know. This question was chosen as it was also asked at *parkrun* registration, allowing direct comparison between pre-*parkrun* participation and post-*parkrun* participation. Given that this was a *parkrun*-specific question, there are no psychometric properties to report for this measure.

This single-item physical activity measure was developed by Milton, Bull [25] and asks*: “In the past week, on how many days have you done a total of 30 minutes or more of physical activity, which was enough to raise your breathing rate. This may include sport, exercise, and brisk walking or cycling for recreation or to get to and from places, but should not include housework or physical activity that may be part of your job.”* Respondents could answer: 0 days, 1 days, 2 days, 3 days, 4 days, 5 days, 6 days, 7 days. This has been validated against the Global Physical Activity Questionnaire in a UK sample of 240 adults [25].

Physical activity was also measured using the International Physical Activity Questionnaire short form (IPAQ-SF) [26]. The IPAQ-SF is a validated, subjective measure of physical activity [27] and was asked as an optional question at the end of the survey, to enable comparison across the different physical activity measures and to give additional insight into the intensity of activity being done. Respondents answered 7 questions on the frequency, intensity (moderate, vigorous, walking, sitting) and duration of physical activity participation over the past 7 days.

#### Perceived impact of running/walking at *parkrun*

The perceived impact of *parkrun* was measured using a question developed by the research team for the purpose of this study: *“Thinking about the impact of parkrun on your health and wellbeing, to what extent has running or walking at parkrun changed:”* Respondents were presented with a list of 15 potential impacts and asked to rate each one on the following 5-point scale: much worse, worse, no impact, better, much better. Examples of impacts included: “*your physical health*”, “*your mental health*”, “*your ability to manage your weight*”, “*your happiness*”, “*the number of new people you meet*” and “*the amount of time you spend with family”* (see Additional file 1 for full list of perceived impacts). The answer choices were displayed in randomised order to help reduce response bias. The final choice was “other” and, if selected, respondents were asked to specify the impact. Given that this was a study-specific question, there are no psychometric properties to report for this measure.

#### *Parkrun* data

Additional data was exported from the *parkrun* database when enough personal details were provided to enable data matching. Additional data matched to responses included the following: postcode provided at *parkrun* registration; date of *parkrun* registration; self-reported physical activity level at registration using the four-week recall question; and total number of *parkruns* completed since registration.

### Data collection

Pilot testing was carried out on a randomly selected sample of 200 UK participants (aged 16 or over). Subsequent power calculations suggested that the survey would have to be sent to the full *parkrun* population to allow segmentation to a sub-sample from socioeconomically deprived areas (derived by postcode) *and* who were previously inactive at registration (less than one bout of activity a week). The survey was distributed between 29th October and 3rd December 2018.

The survey used Qualtrics online survey software [28]. The web link contained an introductory page with a participation information sheet and a confirmation box to indicate it had been read, understood and consent given to be part of the take part. Only people emailed the web link could access the survey. View rate of the survey was not captured. The survey was open for 5 weeks from 29th October 2018 with staggered sending of emails due to email server limitations. Reminders were emailed after 1 week. There were no incentives offered for taking part in the survey.

Questions were asked in the order presented in Additional file 1, with the exception of the International Physical Activity Questionnaire Short Form (IPAQ-SF), which was asked as a final, optional question due to its length and to keep it apart from the other physical activity measures used earlier in the survey. Questions were not randomised, but response choices within some questions were (see Additional file 1).

Adaptive questioning was utilised, such that certain questions were displayed based on answers to previous questions. For example, people who reported being walkers/runners did not see questions about volunteering at *parkrun*. There was a maximum of 47 questions, with an average of 4.3 questions per page and a maximum number of 11 screens (pages) of questions (total question number and page number were shorter depending on how respondents answered questions).

Questions were optional (i.e. non-compulsory) with the exception of the question about *parkrun* participation type (to enable the appropriate questions to be presented to the respondent), one question about long-term health conditions and two questions about life satisfaction and happiness. Respondents could go back and forth within the survey to review or change answers. Upon clicking ‘submit’, answers could not be changed. With consent, partially completed survey responses were saved and data kept for analysis unless the respondent requested removal by contacting the research team.

### Data handling

Survey returns that included identifiers (*parkrun* ID number, name, date of birth, home *parkrun*) were matched, with consent, to *parkrun* registration data for 74% of survey respondents (the remaining 26% did not contain enough information to allow the match). All data was pseudonymised after matching with *parkrun* registration data. Data was handled in accordance with the Data Protection Act 2018 and the General Data Protection Regulation 2018. Data cleaning and analysis was carried out in Microsoft Excel, SPSS (IBM SPSS Statistics 24.0) and MATLAB (version 13.0b, MathWorks, USA).

Duplicate responses were identified by their unique Qualtrics code assigned during the survey and only the latest time-stamped response retained. Responses were excluded if they consented and filled out some or all demographic data but did not fill out any other survey questions to enable analysis. Six respondents were removed either due to abusive comments in free text, because of nonsensical responses, or both. Respondents were not obliged to answer all questions and partially completed surveys were included in the analysis, meaning the sample size varied across each analysis. Cases with missing data on certain variables were omitted from that specific analysis (listwise deletion) and we have reported the relevant sample sizes in all tables.

### Data analysis

Descriptive statistics were used to characterise the respondents and compare them to the total population of *parkrun* registrants from which they were drawn. Sub-sample analyses where then undertaken to compare health and wellbeing, motivation for participation and self-reported benefits of participation between groups defined by socioeconomic deprivation status as well as their self-reported activity level at registration. Respondents from the most socioeconomically deprived areas (IMD quartile 1) are labelled ‘deprived sub-sample’ and those who self-reported as being the least active at *parkrun* registration (i.e. less than one bout a week) are labelled ‘inactive sub-sample’. Respondents from the most socioeconomically deprived areas *and* the least active at registration are labelled ‘deprived/inactive sub-sample’.

For descriptive statistics, we report percent, mean, median and interquartile range (IQR). Data such as age, happiness, life satisfaction, health today, *parkruns* per year, years registered with *parkrun* and the single physical activity question were non-parametric. Group comparisons were carried out using the Mann-Whitney U test. The alpha level used as the criterion for statistical significance in all inferential tests was *p* < 0.05 or lower. Effect sizes were calculated using Cohen’s *d* using a pooled standard deviation with sizes defined as follows: small 0.10; medium 0.5; large 0.8; very large 1.2; huge 2.0.

## Results

### Survey responses

The survey resulted in 100,864 respondents (4.5% participation rate). The following were removed from the analysis: 1) respondents who did not consent (1349); 2) respondents who consented to view the survey but did not answer any questions (37,040); 3) respondents who had registered with *parkrun* but not participated (1787); 4) respondents who identified as *parkrun* volunteers (681), i.e. were not runners or walkers; and 5) respondents who provided invalid responses [7]. The dataset used in this manuscript had 38,071 who identified as runners/walkers and 21,929 who identified as runners/walkers who volunteer, giving a combined data set of 60,000 (2.7% completion rate).

### Demographic characteristics of respondents

Table 1 shows the characteristics of *parkrun* population from its inception on 2nd October 2004 to 3rd December 2018. The mean age was 40.5 years with 51.3% female; 181,561 or 13.1% were from the most deprived areas while 109,296 or 6.6% were previously inactive at registration. They had run or walked approximately 3.7 *parkrun*s per year and been registered with *parkrun* for around 3.5 years.

Table 2 shows that the deprived, inactive and deprived/inactive sub-samples had 4384, 2184 and 237 respondents respectively. The proportion of the full sample who were female was 51.7% (similar to the full *parkrun* population); this increased in the deprived, inactive and deprived/inactive sub-samples to 52.5, 54.8 and 56.1% respectively. The mean age of the full survey sample was older than the *parkrun* population (48.0 ± 13.1 years compared to 40.5 years in the *parkrun* population). Mean age decreased for the deprived, inactive and deprived/inactive sub-samples to 44.3 ± 12.7, 45.6 ± 12.6 and 43.6 ± 12.0 years respectively (all significant at *p* < 0.001 with small to medium effect sizes).

**Table 2:** Data for participants who were runners/walkers and runners/walkers who volunteer. Data in grey-italic indicate numbers < 10. (Percentages may not sum to 100% due to rounding)

**(a) Demographic**	**Sample/sub-sample**				
	**Full sample**		**Deprived sub-sample**	**Inactive sub-sample**	**Deprived / inactive sub-sample**
Survey responses (n)	60,000		4384	2184	237
Proportion female	51.7%		52.5%	54.8%	56.1%
Age (years)					
n	59,618		4377	2183	237
Mean	48.0		44.3 ^z^	45.6 ^z^	43.6 ^z^
Standard deviation	13.1		12.7	12.6	12.0
Effect size			0.29	0.19	0.34
Index of multiple deprivation					
n	46,153		4384	2134	237
Quartile 1	9.5%		100%	11.1%	100%
Quartile 2	20.4%			22.2%	
Quartile 3	30.0%			30.4%	
Quartile 4	40.1%			36.3%	
Physical activity level at registration					
n	42,747		4041	2184	237
Inactive < 1 per week	5.1%		5.9%	100%	100%
Active ≈ 1 per week	11.5%		11.3%		
Active ≈ 2 per week	22.8%		22.5%		
Active ≈ 3 per week	33.8%		34.0%		
Active ≥4 per week	26.9%		26.3%		
Ethnicity					
n	59,340		4342	2167	233
White	96.4%		94.0%	94.9%	93.1%
Black, Asian or Other ethnic background	2.9%		5.3%	4.5%	6.0%
Rather not say	0.8%		0.8%	0.6%	0.9%
Employment status					
n	58,433		4277	2117	229
Full-time paid employment	55.7%		64.6%	59.3%	64.2%
Part-time paid employment	14.0%		11.6%	15.7%	15.3%
Fully retired	12.5%		7.4%	8.1%	4.4%
Self-employed	9.5%		8.0%	8.6%	6.6%
Student	3.1%		3.4%	3.3%	3.1%
Unemployed and not working	1.2%		1.7%	2.1%	3.1%
Other	4.1%		3.4%	2.8%	3.5%
**(b) Health at survey**	**Sample/sub-sample**				
	**Full sample**		**Deprived sub-sample**	**Inactive sub-sample**	**Deprived / inactive sub-sample**
Happiness (0–10)					
n	59,998		4384	2184	237
Mean	7.52		7.35 ^z^	7.26 ^z^	7.11 ^y^
Standard deviation	1.72		1.80	1.79	1.95
Effect size			0.10	0.15	0.24
Life satisfaction (0–10)					
n	59,993		4384	2183	237
Mean	7.76		7.58 ^z^	7.48 ^z^	7.37 ^z^
Standard deviation	1.46		1.54	1.53	1.60
Effect size			0.12	0.19	0.27
Health today (0–100)					
n	57,283		4205	2093	225
Mean	81.0		79.3 ^z^	77.3 ^z^	74.7 ^z^
	12.7		13.7	14.3	15.2
Effect size			0.13	0.29	0.50
**(c) Motives**	**Sample/sub-sample**				
	**Full sample**		**Deprived sub-sample**	**Inactive sub-sample**	**Deprived / inactive sub-sample**
Motives					
n	59,263		4344	2161	234
(Rank) Proportion of n for top 10 motives					
To contribute to my fitness	(1) 56.2%		(1) 52.2%	(1) 50.6%	(2) 45.3%
To improve my physical health	(2) 37.0%		(2) 39.5%	(2) 49.1%	(1) 48.3%
To gain a sense of personal achievement	(3) 26.9%		(3) 26.0%	(4) 25.4%	(5) 25.6%
To get a recorded time for a 5 k	(4) 21.4%		(4) 22.0%	(7) 11.7%	(7) 12.8%
To manage my weight	(5) 19.8%		(5) 21.4%	(3) 29.2%	(3) 32.5%
My friends, family or colleagues encouraged me to	(6) 15.2%		(7) 15.1%	(5) 24.5%	(4) 26.1%
To train for another sport/event	(7) 14.2%		(8) 13.9%	(10) 6.7%	(9) 8.1%
To improve my mental health	(8) 13.0%		(6) 16.8%	(6) 17.1%	(6) 18.8%
To feel part of a community	(9) 11.0%		(9) 11.3%	(9) 6.8%	(10) 6.0%
To spend time outdoors	(10)10.3%		(10)10.2%	(8) 8.2%	(8) 10.3%
**(d) parkrun participation**	**Sample/sub-sample**				
	**Full sample**		**Deprived sub-sample**	**Inactive sub-sample**	**Deprived / inactive sub-sample**
Years registered with *parkrun*					
n	47,701		4300	2184	237
Mean	3.13		2.71 ^z^	2.40 ^z^	2.28 ^z^
SD	2.53		2.30	1.92	1.80
Median	2.61		2.17	1.99	1.84
Q1-Q3	0.94–4.81		0.72–4.20	0.74–3.82	0.68–3.46
Effect size			0.17	0.29	0.34
Total *parkruns* run/walked					
n	45,708		4193	2116	232
Mean	46.0		39.2 ^z^	37.4 ^z^	35.0 ^x^
Standard deviation	61.1		54.7	46.9	48.2
Median	21		17	18	15
Q1-Q3	6–62		5–51	6–50	6–44
Effect size			0.11	0.14	0.18
*Parkruns* run/walked per year					
n	34,211		2942	1447	151
Mean	14.60		14.12 ^x^	15.53 ^y^	14.78
Standard deviation	12.15		12.02	12.50	12.67
Median	11.3		10.7	12.2	11.0
Q1-Q3	4.0–23.3		3.9–22.5	4.4–25.4	3.9–24.1
Effect size			0.04	0.08	0.01
**(e) physical activity at the survey**	**Sample/sub-sample**				
	**Full sample**	**Deprived sub-sample**	**Inactive sub-sample**	**Deprived / inactive sub-sample**	
Single activity question					
n	59,967	4382	2183	236	
Mean	3.59	3.45 ^z^	2.41 ^z^	2.47 ^z^	
Standard deviation	1.77	1.81	1.67	1.71	
Median	3	3	2	2	
Q1 – Q3	2–5	2–5	1–3	1–3	
Effect size		0.08	0.67	0.64	
IPAQ n	45,496	3303	1568	171	
Proportion low or moderate physical activity	35.8%	38.0%	62.2%	59.6%	
Proportion high physical activity (health enhancing)	64.2%	62.0%	37.8%	40.4%	

Mann-Whitney test between full sample and sub-samples: ^x^ *p* < 0.05; ^y^ *p* < 0.01; ^z^ *p* < 0.001

Effect size was calculated using Cohen’s *d* using a pooled standard deviation. Effects are defined as follows: small 0.10; medium 0.5; large 0.8; very large 1.2; huge 2.0

The full sample was 96.4% White with 2.9% from a Black, Asian or other ethnic background; the latter increased for the sub-samples to 6.0% for the deprived/inactive sub-sample. 55.7% of the full sample were in full-time employment with an additional 14.0% part-time and 9.5% self-employed; 12.5% were retired, 3.1% were students and 1.2% were unemployed. The proportion in the sub-samples who were retired decreased (to a minimum of 4.4% for the deprived/inactive sub-sample) while those who were unemployed increased (to 3.1% for the deprived/inactive sub-sample).

Table 2 shows values for happiness, life satisfaction and health for the full sample and sub-samples. Those in the deprived sub-sample reported 2.3% lower happiness than the full sample (7.35 ± 1.80 compared to 7.52 ± 1.72 out of 10; *p* < 0.001; effect size = 0.10) and 2.3% less life satisfaction (7.58 ± 1.54 compared to 7.76 ± 1.46 out of 10; *p <* 0.001; effect size = 0.12). This reduction increased for the inactive sub-sample to 3.3% for happiness (7.26 ± 1.79; *p* < 0.001; effect size = 0.15) and 3.5% for life satisfaction (7.48 ± 1.53; *p* < 0.001; effect size = 0.19). The deprived/inactive sub-sample reported 5.3% less happiness (7.11 ± 1.95; *p* < 0.01; effect size = 0.24) and 4.5% less life satisfaction than the full sample (7.37 ± 1.60; *p* < 0.001; effect size = 0.27). It should be noted that the sample size was small in the latter group (*n* = 237). In England and Wales, national happiness has been reported as 7.53 out of 10 and life satisfaction 7.69 out of 10 [22].

In terms of overall health as measured by the EQ-VAS, those in the deprived sub-sample reported 2.1% lower health scores than the full sample (79.3 ± 13.7 compared to 81.0 ± 12.7 out of 100; *p <* 0.001; effect size = 0.13); those in the inactive sub-sample reported 4.6% lower health scores (77.3 ± 14.3; *p <* 0.001; effect size = 0.29) and the deprived/inactive sub-sample reported the greatest reduction at 7.8% (74.7 ± 15.2; *p <* 0.001; effect size = 0.50) compared to the full sample. It should be noted that there were only 225 respondents in the deprived/inactive sub-group.

### Motives for participating in *parkrun*

Respondents to the survey were asked to select three motives for initially taking part in *parkrun*: the results are shown in Table 2. The first and second most reported motives for the full sample were ‘to contribute to my fitness’ (56.2% of respondents) and ‘to improve my physical health’ (37.0% of respondents). The proportions choosing fitness tended to decrease for the deprived and inactive sub-samples, while the proportions choosing physical health tended to increase. The rankings reversed for the deprived/inactive sub-sample, so that ‘to improve my physical health’ was the first-ranked motive (48.3% of respondents) while ‘to contribute to my fitness’ was the second (45.3% of respondents).

The motive ‘to gain a sense of personal achievement’ was ranked third in the full sample and had a similar proportion of respondents across the sub-samples (25.4 to 26.9%). The fourth ranked motive in the full sample was ‘to get a recorded time for a 5k’ at 21.4%; this reduced to 11.7% for the inactive sub-sample and to 12.8% for the deprived/inactive sub-sample so that it was ranked seventh place. In contrast, the fifth ranked motive for the full sample was ‘to manage my weight’ (19.8%); this moved up to third place for the inactive and deprived/inactive sub-samples (29.2 and 32.5% respectively).

### Participation and physical activity levels

Table 2 shows the frequency of participation in *parkrun*. The full sample was registered for 3.13 ± 2.53 years; all sub-samples were registered more recently than the full sample with the deprived/inactive sub-sample registered for 2.28 ± 1.80 years (*p* < 0.001; effect size = 0.34). The total number of *parkruns* run or walked was highly skewed with the full sample doing a mean of 46.0 ± 61.1 *parkruns* and median of 21 *parkruns*. The sub-samples completed fewer *parkruns* with the deprived/inactive sub-group doing least (35.0 ± 48.2 *parkrun*s; *p* < 0.05; effect size = 0.18). The mean number of *parkruns per year* run or walked by the full sample was 14.6 ± 12.2 and, although the deprived and inactive sub-samples were statistically different (14.1 ± 12.0 and 15.5 ± 12.5 respectively), the effect sizes were small (0.04 and 0.08 respectively).

Comparison of the *parkrun* physical activity question asked at the survey compared to that asked at *parkrun* registration (see Additional file 2) showed that 88.2% of the inactive sub-sample reported an increase in their activity level following *parkrun* participation. A similar increase of 86.5% was found for the deprived/inactive sub-sample. The median number of days of activity for this previously inactive group had increased to 2 days of activity per week.

Table 2 shows findings from the single-item physical activity measure developed by Milton, Bull [24]. The full sample reported doing 3.59 ± 1.77 days of activity per week, while those in the inactive sub-sample reported 2.41 ± 1.67 days per week. Those in the deprived/inactive sub-sample reported a similar value of 2.47 ± 1.71 days of activity. The IPAQ-SF results (Table 2) indicated that 37.8% of the inactive sub-sample and 40.4% of the deprived/inactive sub-sample did physical activity that was vigorous enough to be health enhancing, according to the scoring system provided by IPAQ-SF [26].

### Perceived impact of running or walking at *parkrun*

The reported benefits for the sub-samples are compared with the full sample in Table 3: response counts are shown in the table. All respondents tended to select no impact, better or much better for the 15 perceived impacts of *parkrun*. The proportion selecting worse or much worse was on average 0.5% for the 15 impacts, apart from ‘the amount of time spent with family’ at 6.2%.

**Table 3:** Perceived impact of running or walking at *parkrun* using the question “Thinking about the impact of *parkrun* on your health and wellbeing, to what extent has running or walking at *parkrun* changed:” Allowed responses were ‘much worse, worse, no impact, better, much better’. Proportions are a combined value of ‘better’ and ‘much better’

Reporting ‘better’ or ‘much better’	Full sample	Deprived sub-sample	Inactive sub-sample	Deprived / inactive sub-sample
Your sense of personal achievement				
n	56,276	4131	2071	223
%	90.7%	91.7%	93.4%	93.3%
Your fitness				
n	56,269	4125	2072	223
%	89.3%	91.3%	92.9%	92.4%
Your physical health				
n	56,262	4134	2077	225
%	84.7%	87.0%	88.5%	89.8%
Your happiness				
n	56,217	4126	2068	224
%	78.8%	81.8%	80.8%	83.5%
The amount of time you spend outdoors				
n	56,251	4134	2076	225
%	74.1%	78.7%	82.1%	85.8%
Your enjoyment of competing				
n	56,253	4126	2072	224
%	72.7%	74.2%	70.6%	70.1%
How much you feel part of a community				
n	56,217	4120	2076	225
%	69.7%	70.6%	68.2%	69.8%
Your mental health				
n	56,215	4127	2074	225
%	69.3%	73.9%	72.3%	76.4%
Your confidence				
n	56,225	4132	2075	225
%	61.3%	66.3%	64.0%	70.7%
Your ability to be active in a safe environment				
n	56,193	4122	2072	225
%	59.9%	65.3%	69.3%	72.4%
The number of new people you meet				
n	56,237	4127	2075	225
%	57.5%	58.7%	55.8%	60.9%
Your ability to control your weight				
n	56,208	4124	2074	224
%	52.3%	54.7%	56.3%	54.0%
Your overall lifestyle choices (e.g. diet & smoking)				
n	56,209	4118	2074	224
%	51.8%	56.4%	57.2%	65.2%
The amount of time you spend with friends				
n	56,181	4125	2073	224
%	41.1%	42.4%	41.1%	46.0%
The amount of time you spend with family				
n	56,140	4123	2071	224
%	27.7%	26.2%	31.7%	29.5%

Table 3 shows the proportions of respondents reporting only improvements to the measures since participating in *parkrun*, i.e. a combined value of those reporting ‘better’ and ‘much better’. The data for the full sample shows that, ‘sense of personal achievement’ had the largest proportion of 90.7%. The second highest rated measure was fitness (89.3%) followed by physical health (84.7%), happiness (78.8%) and the amount of time spent outdoors (74.1%). Mental health was improved for 69.3% of respondents and ‘overall lifestyle choices’ improved for 51.8%.

The proportion reporting improvements on the perceived impacts tended to be higher for the deprived sub-sample, higher again for the inactive sub-sample and highest for the deprived/inactive sub-sample. A notable exception to this was ‘enjoyment of competing’ where the proportion decreased from 72.7% for the full sample to 70.6% for the inactive sub-sample and 70.1% for the deprived/inactive sub-sample.

## Discussion

In this self-selected sample of *parkrun* participants, all respondents, irrespective of demographic characteristics and socioeconomic deprivations status, reported diverse benefits from participation in *parkrun* as runners/walkers. Whilst there was response bias in favour of those participating in *parkrun* more frequently, and fewer responses from those from more socioeconomically deprived areas and less active at registration, the scale of the survey ensured that comparison of these sub-groups with the sample as a whole was possible. We were able, for the first time, to compare benefits in those groups who have the greatest theoretical capacity to benefit from participation in *parkrun* with other sub-groups from within the *parkrun* population. This addresses a key priority linked to the achievement of population goals identified in the WHO’s Global Action Plan on Physical Activity [1].

When compared to the full sample, the deprived, inactive and deprived/inactive sub-samples had a larger proportion of females, were younger, less likely to be retired and more likely to be unemployed; they were also more likely to be from a Black, Asian or other ethnic minority background and more likely to report having long-term health conditions. These factors could contribute to the lower happiness, life satisfaction and health score for the sub-samples, which warrants further investigation, especially as more of the deprived and inactive sub-groups reported improvements to health and wellbeing impacts due to *parkrun* compared to the full sample.

The *parkrun* participants (runners/walkers) in our survey who were previously inactive reported an increase in their activity levels from doing less than 1 day of activity per week at registration, to doing on average 2.4 days per week. Thus, in addition to the 15 or so *parkruns* completed per year on average, this would equate to another 111 days per year of physical activity outside *parkrun*; this increases to 115 days per year if they are also from more deprived areas. If the reported increases in physical activity observed here were to be replicated in the full *parkrun* population, then this could have substantial public health value. Given that individuals living in more socioeconomically deprived areas who are physically active may experience better health and quality of life than their neighbours who are less active [15], further research is needed to explore how community physical activity initiatives like *parkrun* can use strategies that promote inclusivity and encourage better representation from currently underrepresented populations.

Whilst the range and magnitude of benefits reported in this study indicate that respondents from across all sub-groups believe running or walking at *parkrun* impacted positively on their health and wellbeing, more of those who were from the most socioeconomically deprived areas, and those least active at registration, reported greater improvements than the full sample. Despite this, their self-reported health and wellbeing was consistently lower than the full sample, reflecting persistent and widely recognised health inequalities.

### Further research to explore factors related to benefits from participation

There is a growing body of qualitative research exploring the motivations for participation in *parkrun* and the positive benefits experienced by those who attend [7–9, 29, 30]. Research has also explored the barriers to participation for specific communities and population groups and the potential for action research in developing inclusive strategies to increase participation by underrepresented groups [11]. Valuable insights could be gleaned from understanding the barriers to participation in community initiatives like *parkrun* among people from more inactive groups, including those from socioeconomically deprived areas. Such research would help build a more nuanced understanding of the factors that underpin participation. Working with communities to understand these challenges is an important step in designing inclusive strategies to promote participation that could potentially translate into important health benefits and contribute to reducing health inequalities.

Further analysis of matched *parkrun* data, using recorded *parkrun* participation as well as survey responses, could be used to explore the complex and bi-directional relationship between frequency of participation and changes in health and fitness (for which recorded *parkrun* completion times may be a proxy) and reported benefits. These relationships may vary for different types of benefit, with some benefits being experienced at lower levels of engagement and frequency of participation than others. It is also likely that overall perceived benefits may be related to the original motivation for participation.

The benefits related to volunteering at *parkrun*, as well as those related to running and walking should also be explored, as there is substantial evidence from previous research that there can be direct and substantial health and wellbeing benefits from volunteering, such as positive impacts on mental and physical health, life satisfaction, social well-being and depression [31]. The potential impact of *parkrun* volunteering, compared to running/walking at *parkrun*, is being explored by the authors separately. There is also potential for *parkrun* and similar community-based events to address current inequalities in both volunteering opportunities and the related benefits [32].

The overall benefits to a community are likely to be much greater than the sum of the benefits reported by individual participants. Wider benefits may include improved perceptions of the local area, increased economic activity if participants use local cafes and shops when attending an event [30], community spirit [29, 33] and linking stakeholders within a community, as seen in the UK’s *parkrun practice* initiative [34]. Previous researchers have used a Social Return of Investment methodology to quantify the wider benefits due to sport [35]. A similar analysis of *parkrun* would allow potential funders, local authorities and those wishing to set up similar interventions to understand their social impact and return on investment.

### Implications for policy and practice

The example of *parkrun* shows that large-scale, mass participation physical activity initiatives could impact positively on the health and wellbeing of participants and have the potential to address health inequalities. It has been assumed that the population groups with lowest levels of physical activity and highest risk of the associated chronic health conditions, who are also more likely to live in more socioeconomically deprived areas, potentially have the most to gain from being more active. However inequalities in personal and environmental resources, including access to transport and free time for recreation at weekends, and other social and cultural barriers to attendance, are reflected in disparities in health behaviours (e.g. recreational physical activity) [36]. In terms of motives, the results of this study imply that those from socioeconomically deprived areas, who were previously inactive, or both are more motivated by their health and improving their lifestyle than fitness, competition or training for other events. *parkrun* and other organisations might consider these factors when starting new community events.

This study shows that if these population groups do participate in recreational physical activity, they do (as might be hoped if not expected) report the highest levels of benefits. Further research is needed into the barriers experienced by people who theoretically have the most to gain from participation.

### Strengths and limitations

The major strength of this study is the size and diversity of the dataset that ensured that, despite the low response rate and response bias expected for an email based online survey [20], the sample had the statistical power to explore variation between sub-groups of participants including those underrepresented in previous research i.e. those least active at registration and those living in the most socioeconomically deprived areas of the UK.

The findings should be interpreted in light of further methodological considerations. The cross-sectional nature of the data (a sub-sample of the *parkrun* population at one snapshot in time) means the associations observed cannot be inferred as causal; many influential factors outside of *parkrun* may have contributed to the positive changes observed. Longitudinal studies are needed to explore how *parkrun* and health and wellbeing interact over time.

The findings should be interpreted with small sub-sample sizes in mind, especially the deprived/inactive sub-sample. The socioeconomic deprivation status of respondents was not studied directly through questions about employment, income etc., but was inferred from IMD derived by the postcode provided at *parkrun* registration. This gave a proxy socioeconomic status measure for the area lived in when the respondent first registered with *parkrun*, rather than specific to the respondent at the time of survey completion. The survey was only available in online format in the English language which may potentially exclude people who had limited internet access or low literacy and digital literacy levels. Future implementation of this survey would benefit from designing, testing and piloting the survey with members of the public, especially those representing underrepresented groups such as people from Black, Asian and other ethnic minority backgrounds and those from areas of higher socioeconomic deprivation.

A further aspect of the survey design worthy of consideration is that a combination of pre-existing, validated survey questions and study-specific questions derived by the research team were used. This was deemed a pragmatic decision to ensure that responses were relevant to *parkrun* participation, but introduces some inconsistency to the methods and potential bias to the findings.

Response bias could also be assessed from the matching of survey responses to *parkrun* registration data available for the full sample. This indicates that the main difference between respondents and *parkrun* participants invited to complete the survey is in the number of *parkrun* events attended (14.5 vs. 3.7 *parkruns* per year). The results therefore relate to a sample that attend *parkrun* more often and that in addition may well have experienced higher levels of perceived benefit, leading in turn to both more frequent attendance and greater motivation to complete a questionnaire on their health and wellbeing in relation to *parkrun* participation.

Given this, we also undertook an analysis of a truncated sub-sample that was more representative of the *parkrun* participant population (*n* = 31,632) where the mean was 3.7 *parkruns* per year (achieved by excluding those who had done more than 8.85 *parkruns* per year; see Additional files 3 and 4). Even in this truncated sample, the benefits of *parkrun* to respondents were similar to the full sample.

## Conclusions

Survey respondents, representing *parkrun* participants with a diverse range of demographic and socioeconomic characteristics and of physical activity levels at *parkrun* registration, reported a wide range of benefits that they credited to *parkrun* participation. Around 9 out of 10 of those who were previously inactive reported increases to their physical activity and similar proportions reported improvements to their physical health and fitness. This proportion increased further for those from socioeconomically deprived areas. The results show that *parkrun* and similar initiatives can introduce large numbers of people from diverse backgrounds to recreational physical activity and impact positively on a high proportion of them. It is important that future research helps identify *how* community initiatives like *parkrun* can better engage with those groups who potentially have most to gain from being more active in order to maximise impact.

## Supplementary Information

**Additional file 1 **Survey details. Variables, outcome measures and questions captured in the *parkrun* Health and Wellbeing survey 2018.

**Additional file 2.** Activity change. Activity at the survey for those who were in the inactive category (less than once per week) at registration.

**Additional file 3 **Data from survey with truncated sample. Data for participants who were runners/walkers and runners/walkers who volunteer for the full sample and a truncated sample who participated in ≤8.85 *parkruns* per year.

**Additional file 4 **Perceived impact with truncated sample. Perceived impact of running or walking at *parkrun* using the question “Thinking about the impact of *parkrun* on your health and wellbeing, to what extent has running or walking at *parkrun* changed”. Allowed responses were ‘much worse, worse, no impact, better, much better’. Data in the table is a combined value for ‘better’ and ‘much better’. Results are compared to a truncated sample who participated in ≤8.85 *parkruns* per year.

## Data Availability

The datasets supporting the conclusions of this article are stored in the Sheffield Hallam University Research Database (SHURDA) for access and in accordance with the Data Protection Act 2018 and the General Data Protection Regulation 2018. In the hope of ensuring the full research potential of the dataset, a copy of the anonymised data will be accessible to researchers for research purposes through the *parkrun* Research Board, as originally outlined in the participant information sheet. Please contact Prof Steve Haake for details about requesting data: s.j.haake@shu.ac.uk.

## Abbreviations

EQ-5D: EuroQol
EQ-VAS: EuroQol Visual Analogue Scale
IMD: Index of Multiple Deprivation
IPAQ-SF: International Physical Activity Questionnaire Short Form
LSOA: Lower Level Super Output Area
UK: United Kingdom
WHO: World Health Organization
