# Supplementary material for: Exploring the benefits of participation in community-based running and walking events: a cross-sectional survey of parkrun participants
Source: BMC Public Health. 2021 Nov 2;21:1978. doi: 10.1186/s12889-021-11986-0 (PMC8561845; doi:10.1186/s12889-021-11986-0)
Supplement: Supplementary file 1 — Additional file 1 Survey details. Variables, outcome measures and questions captured in the parkrun Health and Wellbeing survey 2018. [file 12889_2021_11986_MOESM1_ESM.docx]

**Additional File 1**

Variables, outcome measures and questions captured in the *parkrun* Health and Wellbeing survey 2018.

| Variable and outcome | Question/response options |
| --- | --- |
| Participant details and demographics | - First name - Last name - Date of birth - *parkrun* ID number (allocated at parkrun registration) - Home parkrun - Employment status - Ethnicity - Health condition, illness or disability (a long-term health condition or disability that has lasted or is expected to last 12 months or more) included whether or not the respondent is or has been pregnant in the last 12 months - *parkrun* participation type (whether they participate as a runner/walker only, volunteer only or both) |
| Life satisfaction and happiness | Measures of life satisfaction and happiness: two of the four personal well-being questions asked in the UK's Office of National Statistics Annual Population Survey (Office of National Statistics (ONS), 2018).   - Overall, how satisfied are you with your life nowadays? [Visual analogue scale where 0 is "not satisfied at all", and 10 is "completely satisfied"] - Overall, how happy did you feel yesterday? [Visual analogue scale where 0 is "not at all" and 10 is "completely"] |
| Physical activity level | Three measures of physical activity were used:   1. *parkrun* physical activity question asked at registration: Over the last 4 weeks, how often have you done at least 30 minutes of moderate exercise (enough to raise your breathing rate)? [less than once per week/about once per week/about twice per week/about three times per week/four or more times per week/rather not say/don’t know] 2. International Physical Activity Questionnaire (IPAQ) short version (Craig et al., 2003). The IPAQ is a validated, subjective measure of physical activity (Lee, Macfarlane, Lam, & Stewart, 2011). Respondents answer 7 questions on the frequency, intensity (moderate, vigorous, walking, sitting) and duration of physical activity participation over the past 7 days. 3. Milton, Bull, and Bauman (2011) single-item physical activity measure: In the past week, on how many days have you done a total of 30 minutes or more of physical activity, which was enough to raise your breathing rate. This may include sport, exercise, and brisk walking or cycling for recreation or to get to and from places, but should not include housework or physical activity that may be part of your job. [0 days /1 days/2 days/3 days/4 days/5 days/6 days/7 days] |
| Motives for participation in *parkrun* | - What motivated you to first participate at *parkrun* as a runner or walker? [respondents were asked to select a maximum of three answers out of a possible 21 motives] - What motivated you to first volunteer at *parkrun*? [respondents were asked to select a maximum of three answers out of a possible 27 motives]   The answer choices were displayed in randomised order to help reduce response bias. The final choice was “other” and respondents were asked to specify their motive. |
| Health status | - EQ-5D-5L (Brooks & Group, 1996) characterises health on five dimensions (mobility, self-care, ability to undertake usual activities, pain, anxiety/depression) [five levels: no problems, slight problems, moderate problems, severe problems, extreme problems]. - EQ-5D-5L visual analogue scale (VAS) asked respondents to rate their health today on a scale of 0-100, where 0 is the “worst health you can imagine” and 100 is the “best health you can imagine”. Permission was granted by EuroQol Research Foundation for its use. - In the last 12 months, how many days work have you lost to sickness? (if applicable) |
| Healthcare usage | - How many times in the last 12 months have you: i) been in contact with your GP? ii) Had contact with any (NHS) medical specialists, other than your GP?, iii) Had contact with any medical specialist outside of the (NHS*), iv) Been admitted to hospital, v) Been to A&E, and vi) Been an inpatient (the number days you have stayed in hospital while having treatment) |
| Mental wellbeing | - The Short Warwick-Edinburgh Mental Well-being scale (SWEMWBS) (Tennant et al., 2007). Experience of; feeling optimistic, feeling useful, feeling relaxed, dealing with problems well, thinking clearly, feeling close to people, able to make up my own mind about things [none of time/rarely/some of the time/often/all of the time] |
| Perceived impact of *parkrun* | - Thinking about the impact of *parkrun* on your health and wellbeing, to what extent has running or walking at *parkrun* changed: [list of 15 impacts: much worse/worse/no impact/better/much better] - Thinking about the impact of *parkrun* on your health and wellbeing, to what extent has volunteering at *parkrun* changed: [list of 18 impacts: much worse/worse/no impact/better/much better] - To what extent has running or walking at *parkrun* changed your ability to manage your health condition, disability or illness? [much worse/worse/no impact/better/much better] - To what extent has volunteering at *parkrun* changed your ability to manage your health condition, disability or illness? [much worse/worse/no impact/better/much better]   The answer choices were displayed in randomised order to help reduce response bias. The final choice was “other” and respondents were asked to specify any other impact they have experienced. |
| Social interactions and relationships | - Who do you usually interact with at *parkrun*? i.e., those you attend and communicate with at *parkrun* [list of 11 options]. - In terms of relationships, what opportunities has *parkrun* opened up for you? [list of 9 options].   The answer choices were displayed in randomised order to help reduce response bias. The final choice for both questions was “other” and respondents were asked to specify the other people they usually interact with at parkrun. |
| Open text | At the end of the survey, respondents were invited to add any further comments about the impact of parkrun on their health and wellbeing. |
| Additional data retrieved from the *parkrun* database, with consent | - Date of *parkrun* registration - Gender - Postcode - Self-reported physical activity level at registration - Total number of *parkruns* completed i) as a runner/walker and ii) as a volunteer - Fastest *parkrun* time to date - Average *parkrun* time to date - Number of *parkruns* completed to date per year since registration |

UK Survey - parkrun health and wellbeing survey - Final

Start of Block: Default Block

Q1


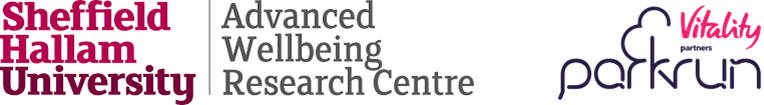


|  |  |
| --- | --- |

Q2 **parkrun health & wellbeing survey** You are invited to take part in the parkrun health and wellbeing survey. In this survey, parkrun and Sheffield Hallam University are asking about your health and wellbeing to better understand the benefits of taking part in 5k parkrun events.
  
 This survey will take around 10 minutes to complete, but take as much time as you need. You will be asked a range of questions about your health and wellbeing. If required, you can ask someone to complete it on your behalf. You can choose not to answer some questions by moving onto the next question. Compulsory questions are marked with an asterisk *.   
 Taking part in this survey is your choice. If you decide to take part, you may leave the survey at any time, but your answers will be saved unless you request to withdraw them.
  
 By completing this survey you agree for us to access your parkrun registration details and parkrun data. All your parkrun data and answers to this survey will be anonymised. 
  
 Please [click here](https://shusls.eu.qualtrics.com/CP/File.php?F=F_8kQmjfvBWO9HC0B%22%20target=%22_blank) to read the full participant information sheet.
  
 For questions or concerns about this survey, or to request an alternative digital format, please contact the research team: [parkrunHWBSurvey@shu.ac.uk](mailto:parkrunHWBSurvey@shu.ac.uk?subject=parkrun%20HWB%20Survey%20UK%202018)   **Giving consent to take part in this survey** I have read (or someone has read to me) this information and the participant information sheet, and I understand that I am being asked to complete a survey about my health and wellbeing. I am aware that I may be contacted again by Sheffield Hallam University or parkrun. I consent to my registration details being used and understand that none of my data will be used or shared in a way that can identify me personally. I agree for my anonymised parkrun data to be used for research purposes. I voluntarily agree to take part.

- I consent to participate in this study
- I DO NOT consent to participate in this study

Skip To: End of Survey If The parkrun health and wellbeing surveyYou are invited to take part in the parkrun health and well = I DO NOT consent to participate in this study

Q3 **About you**

- * First Name: ________________________________________________
- * Last Name: ________________________________________________

Q4
* Date of birth

Q5 **parkrun ID**
Please insert your **parkrun ID**, if you know it (please enter the **number** after A on your parkrun barcode - **do not include the letter A**).

________________________________________________________________

Q6 * Home parkrun

- Complete list of UK parkruns (incl. junior parkruns).

Q7
**Employment status**
Which of the following best describes your current employment status?

- Full-time paid employment
- Part-time paid employment
- Full-time home maker
- Employed, but currently on sick leave
- Employed, but currently on maternity/paternity leave
- Self-employed
- Unemployed and not working
- Fully retired
- Retired, but still in paid employment
- Student
- Other ________________________________________________
- I'd rather not say

Q8 **Ethnicity**
Which of the following best describes your ethnicity?

- White: English/Welsh/Scottish/Northern Irish/British
- White: Irish
- White: Irish Traveller
- Other White background
- White and Black Caribbean
- White and Black African
- White and Asian
- Other Mixed/Multiple ethnic background
- Indian
- Pakistani
- Bangladeshi
- Chinese
- Other Asian background
- African
- Caribbean
- Other Black/African/Caribbean background
- Arab
- Other ethnic group
- I'd rather not say

Q9 * **Health condition, disability or illness** Are your day-to-day activities limited because of a health condition or disability which has lasted, or is expected to last, at least 12 months? Include conditions related to old age, sensory deficits, mobility problems, developmental conditions, learning impairments and mental health.

- Yes, limited a lot
- Yes, limited a little
- No
- Don't know or would rather not say

Display This Question:

If * Health condition, disability or illness Are your day-to-day activities limited because of a hea... = Yes, limited a lot

Or * Health condition, disability or illness Are your day-to-day activities limited because of a hea... = Yes, limited a little

Q10 * What is your health condition, disability or illness? 
  
Please select all that apply.

- ADHD - Attention Deficit Hyperactivity Disorder
- Alcohol or Drug Addiction
- Allergies (any that limit your day-to-day activity)
- Alzheimer's or dementia
- Anxiety Disorder
- Arrhythmia (abnormal heart rate) or Atrial Fibrillation (irregular heart rate)
- Arthritis
- Asthma
- Autism and Asperger's
- Bipolar Disorder
- Cancer (please specify in next question)
- Carpal Tunnel Syndrome
- Chronic Migraines
- Chronic Pain
- COPD (chronic obstructive pulmonary disease) and Emphysema
- Coronary Artery Disease (including angina, peripheral vascular disease)
- Crohn's Disease
- Degenerative Disc Disease
- Depression
- Disorders of the Spine
- Epilepsy or seizure disorder
- Fibromyalgia
- Gout
- Hearing loss or impairment
- Heart Failure
- Hepatitis
- High Blood Pressure (Hypertension)
- Interstitial Cystitis
- Irritable Bowel Syndrome
- Kidney failure or Chronic kidney disease
- Learning disability (e.g., dyslexia, dyspraxia)
- Liver Disease
- Lupus, or Systemic Lupus Erythematosus (SLE)
- Lyme Disease
- Multiple Sclerosis (MS)
- Neuropathy, Peripheral Neuropathy
- Obesity
- Organic Mental Disorders (including Organic Brain Syndrome)
- Osteoporosis
- Panic Attacks
- Parkinson's Disease
- Post Traumatic Stress Disorder (PTSD)
- Reflex Sympathetic Dystrophy (RSD)
- Rheumatoid Arthritis
- Ruptured Disc
- Schizophrenia
- Scoliosis
- Sleep Apnoea
- Stroke (TIA, CVA; Cerebrovascular Accident)
- Thyroid gland disorder
- Traumatic Brain Injury (TBI)
- Type 1 Diabetes
- Type 2 Diabetes
- Ulcerative Colitis
- Venous Thromboembolism (DVT; Deep Venous Thrombosis & Pulmonary Embolism)
- Vision loss or impairment
- Other (please specify in next question)

Display This Question:

If * What is your health condition, disability or illness?    Please select all that apply. = Cancer (please specify in next question)

Or * What is your health condition, disability or illness?    Please select all that apply. = Other (please specify in next question)

Q11 If you ticked "cancer" or "other", please specify your condition, disability or illness:

________________________________________________________________

Q12 Have you been pregnant within the last 12 months?

- Yes
- No
- Would rather not say

Q13 * **parkrun participation**
Choose one option that best describes your current participation at parkrun:

- Runner or walker only
- Volunteer only
- Runner or walker and volunteer
- Registered but not yet participated

|  |
| --- |

Q14 * Life satisfaction & happiness  
Overall, how satisfied are you with your life nowadays?

 Where 0 is 'not at all satisfied' and 10 is 'completely satisfied'. 

 Please enter a number in the box below to indicate how satisfied you are with your life nowadays.

- Satisfaction nowadays = ________________________________________________


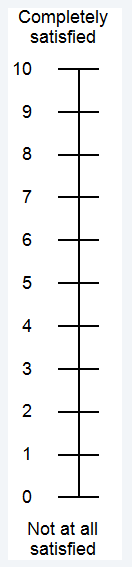


Q15 How happy did you feel yesterday?

 Where 0 is 'not at all happy' and 10 is 'completely happy'.

 Please enter a number in the box below to indicate how happy you felt yesterday.

- Happiness yesterday = ________________________________________________


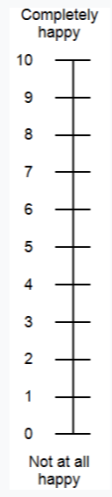


Q16 **Physical activity**
 We are interested in the physical activity parkrunners do as part of their everyday lives. 

 Over the last 4 weeks, how often have you done at least 30 minutes of moderate exercise (**enough to raise your breathing rate**)?

- Less than once per week
- About once per week
- About twice per week
- About three times per week
- Four or more times per week
- Rather not say/don't know

Q17 In the **past week**, on how many days have you done a total of 30 minutes or more of physical activity, which was **enough to raise your breathing rate**. This may include sport, exercise, and brisk walking or cycling for recreation or to get to and from places, but should not include housework or physical activity that may be part of your job.

- 0 days
- 1 days
- 2 days
- 3 days
- 4 days
- 5 days
- 6 days
- 7 days

Display This Question:

If * parkrun participation Choose one option that best describes your current participation at parkrun: = Runner or walker only

Or * parkrun participation Choose one option that best describes your current participation at parkrun: = Runner or walker and volunteer

|  |  |  |
| --- | --- | --- |

Q18 **Motives for running or walking at parkrun**
What motivated you **to first** participate at parkrun as a runner or walker? 
Please tick a **maximum of 3**.

- to improve my physical health
- to improve my mental health
- to improve or manage my health condition, disability or illness
- to manage my weight
- to improve my happiness
- to meet new people
- to spend time with friends
- to spend time with family
- a health professional advised me to
- to compete with others
- to spend time outdoors
- to be active in a safe environment
- to get a recorded time for a 5k
- my friends, family or colleagues encouraged me to
- it was part of a 'couch to 5k' programme
- to feel part of a community
- to gain a sense of personal achievement
- to train for another sport/event
- to raise money for charity
- to contribute to my fitness
- Other (please specify) ________________________________________________

*Answer choices are randomised

Display This Question:

If * parkrun participation Choose one option that best describes your current participation at parkrun: = Runner or walker and volunteer

Or * parkrun participation Choose one option that best describes your current participation at parkrun: = Volunteer only

|  |  |  |
| --- | --- | --- |

Q19 **Motives for volunteering at parkrun**
What motivated you **to first** volunteer at parkrun?
Please tick a **maximum of 3**.

- to improve my physical health
- to improve my mental health
- to improve or manage my health condition, disability or illness
- to improve my happiness
- to meet new people
- to spend time with friends
- to spend time with family
- a health professional advised me to
- to spend time outdoors
- my friends, family or colleagues encouraged me to
- it was part of a volunteering programme or course (e.g. Duke of Edinburgh)
- to feel part of a community
- to gain recognition for my accomplishments
- to help people
- to fulfil a moral duty
- to work with a team of people
- to improve my CV / employability
- as a parkrunner, I felt obliged to volunteer
- to improve my confidence
- it was a good use of my time
- to develop my skills
- to gain a sense of personal achievement
- to contribute to my fitness
- Other (please specify) ________________________________________________
- unable to walk or run (e.g. due to injury, illness or health condition)
- wanted a rest / recovery day
- to give something back to the community

*Answer choices are randomised

| Page Break |  |
| --- | --- |

Q20 **Health** 
 Please click the ONE box that best describes your health TODAY
 
**MOBILITY**

- I have no problems in walking about
- I have slight problems in walking about
- I have moderate problems in walking about
- I have severe problems in walking about
- I am unable to walk about

Q21
© EuroQol Research Foundation. EQ-5D™ is a trade mark of the EuroQol Research Foundation

| Page Break |  |
| --- | --- |

Q22 Please click ONE box that best describes your health TODAY
 **SELF-CARE**

- I have no problems washing or dressing myself
- I have slight problems washing or dressing myself
- I have moderate problems washing or dressing myself
- I have severe problems washing or dressing myself
- I am unable to wash or dress myself

Q23
© EuroQol Research Foundation. EQ-5D™ is a trade mark of the EuroQol Research Foundation

| Page Break |  |
| --- | --- |

Q24 Please click ONE box that best describes your health TODAY   **USUAL ACTIVITIES** *(e.g. work, study, housework, family or leisure activities)*

- I have no problems doing my usual activities
- I have slight problems doing my usual activities
- I have moderate problems doing my usual activities
- I have severe problems doing my usual activities
- I am unable to do my usual activities

Q25
© EuroQol Research Foundation. EQ-5D™ is a trade mark of the EuroQol Research Foundation

| Page Break |  |
| --- | --- |

Q26 Please click ONE box that best describes your health TODAY   **PAIN / DISCOMFORT**

- I have no pain or discomfort
- I have slight pain or discomfort
- I have moderate pain or discomfort
- I have severe pain or discomfort
- I have extreme pain or discomfort

Q27
© EuroQol Research Foundation. EQ-5D™ is a trade mark of the EuroQol Research Foundation

| Page Break |  |
| --- | --- |

Q28 Please click the ONE box that best describes your health TODAY
 
**ANXIETY / DEPRESSION**

- I am not anxious or depressed
- I am slightly anxious or depressed
- I am moderately anxious or depressed
- I am severely anxious or depressed
- I am extremely anxious or depressed

Q29
© EuroQol Research Foundation. EQ-5D™ is a trade mark of the EuroQol Research Foundation

| Page Break |  |
| --- | --- |

Q30
We would like to know how good or bad your health is TODAY. 

This scale is numbered from 0 to 100. 
 
100 means the **best** health you can imagine. 
0 means the **worst** health you can imagine. 
 
*Please enter a number in the box below to indicate how your health is TODAY.*

- Your health today = ________________________________________________


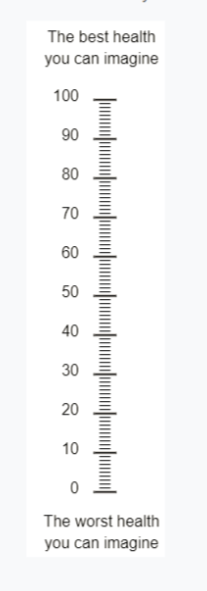


Q31
© EuroQol Research Foundation. EQ-5D™ is a trade mark of the EuroQol Research Foundation

| Page Break |  |
| --- | --- |

Q32 In the last 12 months, how many days work have you lost to sickness? (if applicable)

________________________________________________________________

Q33 How many times in the last 12 months have you:

- Been in contact with your GP _________________________________________
- Had contact with any NHS medical specialists, other than your GP _______________
- Had contact with any medical specialist outside of the NHS ______________________
- Been admitted to hospital _______________________________________________
- Been to A&E ________________________________________________
- Been an inpatient (the number days you have stayed in hospital while having treatment) ________________________________________________

| Page Break |  |
| --- | --- |

Q34 **Mental wellbeing**
Below are some statements about feelings and thoughts. Please tick the box that describes your experience of each **over the last 2 weeks**.

|  | None of the time | Rarely | Some of the time | Often | All of the time |
| --- | --- | --- | --- | --- | --- |
| I’ve been feeling optimistic about the future |  |  |  |  |  |
| I’ve been feeling useful |  |  |  |  |  |
| I’ve been feeling relaxed |  |  |  |  |  |
| I’ve been dealing with problems well |  |  |  |  |  |
| I’ve been thinking clearly |  |  |  |  |  |
| I’ve been feeling close to other people |  |  |  |  |  |
| I’ve been able to make up my own mind about things |  |  |  |  |  |

| Page Break |  |
| --- | --- |

Display This Question:

If * parkrun participation Choose one option that best describes your current participation at parkrun: = Runner or walker only

Or * parkrun participation Choose one option that best describes your current participation at parkrun: = Runner or walker and volunteer

Q35 **The impact of running or walking at parkrun**
 Thinking about the impact of parkrun on your health and wellbeing, to what extent has running or walking at parkrun changed:

|  | Much worse | Worse | No impact | Better | Much better |
| --- | --- | --- | --- | --- | --- |
| your physical health |  |  |  |  |  |
| your mental health |  |  |  |  |  |
| your ability to control your weight |  |  |  |  |  |
| your happiness |  |  |  |  |  |
| the number of new people you meet |  |  |  |  |  |
| the amount of time you spend with friends |  |  |  |  |  |
| the amount of time you spend with family |  |  |  |  |  |
| your enjoyment of competing |  |  |  |  |  |
| the amount of time you spend outdoors |  |  |  |  |  |
| your ability to be active in a safe environment |  |  |  |  |  |
| how much you feel part of a community |  |  |  |  |  |
| your sense of personal achievement |  |  |  |  |  |
| your overall lifestyle choices (e.g. diet and smoking) |  |  |  |  |  |
| your confidence |  |  |  |  |  |
| your fitness |  |  |  |  |  |
| other (please specify) |  |  |  |  |  |

*Answer choices are randomised

Display This Question:

If * parkrun participation Choose one option that best describes your current participation at parkrun: = Runner or walker and volunteer

Or * parkrun participation Choose one option that best describes your current participation at parkrun: = Volunteer only

Q36 **The impact of volunteering at parkrun**
Thinking about the impact of parkrun on your health and wellbeing, to what extent has volunteering at parkrun changed:

|  | Much worse | Worse | No impact | Better | Much better |
| --- | --- | --- | --- | --- | --- |
| your physical health |  |  |  |  |  |
| your mental health |  |  |  |  |  |
| your happiness |  |  |  |  |  |
| the number of new people you meet |  |  |  |  |  |
| the amount of time you spend with friends |  |  |  |  |  |
| the amount of time you spend with family |  |  |  |  |  |
| the amount of time you spend outdoors |  |  |  |  |  |
| how much you feel part of a community |  |  |  |  |  |
| your ability to gain recognition for your accomplishments |  |  |  |  |  |
| your ability to help people |  |  |  |  |  |
| your ability to fulfil moral duties |  |  |  |  |  |
| your ability to work with a team of people |  |  |  |  |  |
| your CV / employability |  |  |  |  |  |
| your confidence |  |  |  |  |  |
| your skills |  |  |  |  |  |
| your sense of personal achievement |  |  |  |  |  |
| your fitness |  |  |  |  |  |
| other (please specify) |  |  |  |  |  |

Display This Question:

If * parkrun participation Choose one option that best describes your current participation at parkrun: = Runner or walker only

Or * parkrun participation Choose one option that best describes your current participation at parkrun: = Runner or walker and volunteer

And If

* Health condition, disability or illness Are your day-to-day activities limited because of a hea... = Yes, limited a lot

Or * Health condition, disability or illness Are your day-to-day activities limited because of a hea... = Yes, limited a little

Q37 * To what extent has **running or walking** at parkrun changed your ability to manage your health condition, disability or illness?

- Much worse
- Worse
- No effect
- Better
- Much better

Display This Question:

If * parkrun participation Choose one option that best describes your current participation at parkrun: = Runner or walker and volunteer

Or * parkrun participation Choose one option that best describes your current participation at parkrun: = Volunteer only

And If

* Health condition, disability or illness Are your day-to-day activities limited because of a hea... = Yes, limited a lot

Or * Health condition, disability or illness Are your day-to-day activities limited because of a hea... = Yes, limited a little

Q38 * To what extent has **volunteering** at parkrun changed your ability to manage your health condition, disability or illness?

- Much worse
- Worse
- No effect
- Better
- Much better

| Page Break |  |
| --- | --- |

Display This Question:

If * parkrun participation Choose one option that best describes your current participation at parkrun: != Registered but not yet participated

|  |  |
| --- | --- |

Q39
**Social bonds and relationships**
Who do you usually interact with at parkrun? i.e., those you attend and communicate with at parkrun.
 
Please tick all that apply.

- Family
- Friends
- Spouse / Partner
- Neighbours
- Work colleagues
- Members of my sports club (e.g. running or walking club)
- Members of my non-sports club, organisation or group
- Strangers
- Other parkrun participants
- I do not interact with anyone
- Other (please specify) ________________________________________________

*Answer choices are randomised

Display This Question:

If * parkrun participation Choose one option that best describes your current participation at parkrun: != Registered but not yet participated

|  |  |
| --- | --- |

Q40 In terms of relationships, what opportunities has parkrun opened up for you? 
 
Please tick all that apply.

- I have met new people of a similar background
- I have met new people of a different background
- I feel closer to my existing friends or family
- I feel part of my community
- I have joined a sports club (e.g. running or walking club)
- I have joined a non-sports club, organisation or group
- It has made no difference to me
- It has allowed me to spend time on my own
- Other (please specify) ________________________________________________

*Answer choices are randomised

Q41 If there is anything else you would like to mention about the impact of parkrun on your health and wellbeing, please insert your comments here.

________________________________________________________________

________________________________________________________________

________________________________________________________________

________________________________________________________________

________________________________________________________________

Q42 * **You have nearly reached the end of the survey**
 Are you willing to answer some more questions about your physical activity in the last 7 days?

- Yes
- No

Skip To: End of Survey If * You have nearly reached the end of the survey Are you willing to answer some more questions abo... = No

|  |  |
| --- | --- |

Q43
These questions will ask you about the time you spent being physically active in the last 7 days. Please answer each question even if you do not consider yourself to be an active person. 
 
Please think about the activities you do at work, as part of your house and yard work, to get from place to place, and in your spare time for recreation, exercise or sport.

 Think about all the **vigorous activities** that you did in the last 7 days. Vigorous physical activities refer to activities that take hard physical effort and make you breathe much harder than normal. Think only about those physical activities that you did for at least 10 minutes at a time.

 During the last 7 days, on how many days did you do **vigorous physical activities** like heavy lifting, digging, aerobics, or fast bicycling?

- No vigorous physical activities ________________________________________________
- 1 day
- 2 days
- 3 days
- 4 days
- 5 days
- 6 days
- 7 days

Display This Question:

If These questions will ask you about the time you spent being physically active in the last 7 days.... != No vigorous physical activities

|  |
| --- |

Q44 How much time did you usually spend doing **vigorous** physical activities on one of those days?

- Hours per day ________________________________________________
- Minutes per day ________________________________________________
- Don't know/not sure

|  |  |
| --- | --- |

Q45 Think about all the **moderate** activities that you did in the **last 7 days**.  **Moderate** activities refer to activities that take moderate physical effort and make you breathe somewhat harder than normal. Think only about those physical activities that you did for at least 10 minutes at a time.
 
During the **last 7 days**, on how many days did you do **moderate** physical activities like carrying light loads, bicycling at a regular pace, or doubles tennis?  Do not include walking.

- No moderate physical activities
- 1 day ________________________________________________
- 2 days
- 3 days
- 4 days
- 5 days
- 6 days
- 7 days

Display This Question:

If Think about all the moderate activities that you did in the last 7 days.  Moderate activities ref... != No moderate physical activities

|  |
| --- |

Q46 How much time did you usually spend doing **moderate** physical activities on one of those days?

- Hours per day ________________________________________________
- Minutes per day ________________________________________________
- Don't know/not sure

|  |  |
| --- | --- |

Q47
Think about the time you spent **walking** in the **last 7 days**.  This includes at work and at home, walking to travel from place to place, and any other walking that you have done solely for recreation, sport, exercise, or leisure.
 
During the **last 7 days**, on how many days did you **walk** for at least 10 minutes at a time?

- No walking
- 1 day ________________________________________________
- 2 days
- 3 days
- 4 days
- 5 days
- 6 days
- 7 days

Display This Question:

If Think about the time you spent walking in the last 7 days.  This includes at work and at home, wa... != No walking

Q48 How much time did you usually spend **walking** on one of those days?

- Hours per day ________________________________________________
- Minutes per day ________________________________________________
- Don't know/not sure

Q49
The last question is about the time you spent **sitting** on weekdays during the **last 7 days**.  Include time spent at work, at home, while doing course work and during leisure time.  This may include time spent sitting at a desk, visiting friends, reading, or sitting or lying down to watch television.

 During the **last 7 days**, how much time did you spend **sitting** on a **week day**?

- Hours per day ________________________________________________
- Minutes per day ________________________________________________
- Don't know/not sure

End of Block: Default Block
