# Supplementary material for: Exploring the benefits of participation in community-based running and walking events: a cross-sectional survey of parkrun participants
Source: BMC Public Health. 2021 Nov 2;21:1978. doi: 10.1186/s12889-021-11986-0 (PMC8561845; doi:10.1186/s12889-021-11986-0)
Supplement: Supplementary file 2 — Additional file 2. Activity change. Activity at the survey for those who were in the inactive category (less than once per week) at registration. [file 12889_2021_11986_MOESM2_ESM.docx]

**Additional File 2**

Physical activity at the survey for those who were in the inactive category (less than once per week) at registration.

| **Activity category at survey** | **Inactive** | **Deprived / Inactive** | **Inactive** | **Deprived / Inactive** |
| --- | --- | --- | --- | --- |
| **<1** | 258 | 32 | 11.7% | 13.5% |
| **≈1** | 460 | 52 | 21.0% | 21.9% |
| **≈2** | 566 | 52 | 25.9% | 21.9% |
| **≈3** | 537 | 66 | 24.7% | 27.8% |
| **≥4** | 358 | 35 | 16.6% | 14.8% |
| **Total** | 2,179 | 237 | 100% | 100% |
| **Increased** | 1,921 | 205 | 88.2% | 86.5% |
| **Median** |  |  | ≈2 | ≈2 |
| **Mode** |  |  | ≈2 | ≈3 |
