# Supplementary material for: Exploring the benefits of participation in community-based running and walking events: a cross-sectional survey of parkrun participants
Source: BMC Public Health. 2021 Nov 2;21:1978. doi: 10.1186/s12889-021-11986-0 (PMC8561845; doi:10.1186/s12889-021-11986-0)
Supplement: Supplementary file 3 — Additional file 3 Data from survey with truncated sample. Data for participants who were runners/walkers and runners/walkers who volunteer for the full sample and a truncated sample who participated in ≤8.85 parkruns per year. [file 12889_2021_11986_MOESM3_ESM.docx]

**Additional File 3**

Data for participants who were runners/walkers and runners/walkers who volunteer for the full sample (see also Table 2) and a truncated sample who participated in ≤ 8.85 parkruns per year. Data in grey-italic indicate numbers <10.

| **(a) Demographic** | **Full sample/sub-sample** | | | | **Truncated sample/sub-sample** | | | |  |
| --- | --- | --- | --- | --- | --- | --- | --- | --- | --- |
|  | **Full sample** | **Deprived sub-sample** | **Inactive sub-sample** | **Deprived / inactive sub-sample** | **Full sample** | **Deprived sub-sample** | **Inactive sub-sample** | **Deprived / inactive sub-sample** | |
| Survey responses (n) | 60,000 | 4,384 | 2,184 | 237 | 31,632 | 1,868 | 789 | 94 | |
| Proportion female | 51.7% | 52.5% | 54.8% | 56.1% | 55.2% | 56.4% | 59.7% | 58.5% | |
| Age (years) n | 59,618 | 4,377 | 2,183 | 237 | 18,168 | 1,862 | 788 | 237 | |
| Mean | 48.0 | 44.3 | 45.6 | 43.6 | 46.9 | 42.8 | 43.6 | 44.0 | |
| Standard deviation | 13.1 | 12.7 | 12.6 | 12.0 | 13.3 | 12.6 | 12.8 | 12.6 | |
| Effect size |  | 0.29 | 0.19 | 0.34 |  | 0.31 | 0.25 | 0.22 | |
| Index of multiple deprivation n | 46,153 | 4,384 | 2,134 | 237 | 18,709 | 1,868 | 766 | 94 | |
| Quartile 1 | 9.5% | 100% | 11.1% | 100% | 10.0% | 100% | 12.3% | 100% | |
| Quartile 2 | 20.4% |  | 22.2% |  | 20.4% |  | 23.0% |  | |
| Quartile 3 | 30.0% |  | 30.4% |  | 29.5% |  | 28.9% |  | |
| Quartile 4 | 40.1% |  | 36.3% |  | 40.1% |  | 35.9% |  | |
| Physical activity level at registration n | 42,747 | 4,041 | 2,184 | 237 | 16,925 | 1,679 | 789 | 94 | |
| Inactive <1 per week | 5.1% | 5.9% | 100% | 100% | 4.7% | 5.6% | 100% | 100% | |
| Active ≈ 1 per week | 11.5% | 11.3% |  |  | 10.4% | 11.8% |  |  | |
| Active ≈ 2 per week | 22.8% | 22.5% |  |  | 21.6% | 21.4% |  |  | |
| Active ≈ 3 per week | 33.8% | 34.0% |  |  | 34.1% | 33.4% |  |  | |
| Active ≥ 4 per week | 26.9% | 26.3% |  |  | 29.4% | 27.8% |  |  | |
| Ethnicity n | 59,340 | 4,342 | 2,167 | 233 | 31,530 | 1,850 | 780 | 91 | |
| White | 96.4% | 94.0% | 94.9% | 93.1% | 96.1% | 93.1% | 94.5% | 92.3% | |
| Black, Asian or Other ethnic background | 2.9% | 5.3% | 4.5% | 6.0% | 3.1% | 6.0% | 5.1% | *6.6%* | |
| Rather not say | 0.8% | 0.8% | 0.6% | 0.9% | 0.8% | 0.9% | 0.4% | *1.1%* | |
| Employment status n | 58,433 | 4,277 | 2,117 | 229 | 31,110 | 1,826 | 761 | 91 | |
| Full-time paid employment | 55.7% | 64.6% | 59.3% | 64.2% | 56.2% | 64.8% | 58.7% | 57.1% | |
| Part-time paid employment | 14.0% | 11.6% | 15.7% | 15.3% | 14.1% | 11.2% | 15.9% | 16.5% | |
| Fully retired | 12.5% | 7.4% | 8.1% | 4.4% | 11.3% | 6.3% | 7.2% | *3.3%* | |
| Self-employed | 9.5% | 8.0% | 8.6% | 6.6% | 9.6% | 8.8% | 9.1% | 12.1% | |
| Student | 3.1% | 3.4% | 3.3% | 3.1% | 3.6% | 3.6% | 4.7% | *4.4%* | |
| Unemployed and not working | 1.2% | 1.7% | 2.1% | 3.1% | 1.2% | 1.5% | 2.0% | *1.1%* | |
| Other | 4.1% | 3.4% | 2.8% | 3.5% | 4.0% | 3.9% | 2.4% | *5.5%* | |
|  |  |  |  |  |  |  |  |  | |
| **(b) Health at survey** | **Full sample/sub-sample** | | | | **Truncated sample/sub-sample** | | | |  |
|  | **Full sample** | **Deprived sub-sample** | **Inactive sub-sample** | **Deprived / inactive sub-sample** | **Full sample** | **Deprived sub-sample** | **Inactive sub-sample** | **Deprived / inactive sub-sample** | |
| Happiness (0 – 10) n | 59,998 | 4,384 | 2,184 | 237 | 31,994 | 1,868 | 789 | 94 | |
| Mean | 7.52 | 7.35 | 7.26 | 7.11 | 7.49 | 7.32 | 7.10 | 7.16 | |
| Standard deviation | 1.72 | 1.80 | 1.79 | 1.95 | 1.75 | 1.80 | 1.91 | 2.22 | |
| Effect size |  | 0.10 | 0.15 | 0.24 |  | 0.10 | 0.22 | 0.19 | |
| Life satisfaction (0 – 10) n | 59,993 | 4,384 | 2,183 | 237 | 31,993 | 1,868 | 789 | 94 | |
| Mean | 7.76 | 7.58 | 7.48 | 7.37 | 7.72 | 7.52 | 7.29 | 7.27 | |
| Standard deviation | 1.46 | 1.54 | 1.53 | 1.60 | 1.49 | 1.56 | 1.64 | 1.80 | |
| Effect size |  | 0.12 | 0.19 | 0.27 |  | 0.13 | 0.29 | 0.30 | |
| Health today (0 – 100) n | 57,283 | 4,205 | 2,093 | 225 | 30,313 | 1,784 | 752 | 90 | |
| Mean | 81.0 | 79.3 | 77.3 | 74.7 | 80.6 | 78.7 | 75.6 | 73.3 | |
| Standard deviation | 12.7 | 13.7 | 14.3 | 15.2 | 13.1 | 14.1 | 15.0 | 16.5 | |
| Effect size |  | 0.13 | 0.29 | 0.50 |  | 0.14 | 0.38 | 0.56 | |

| **(c) Motives** | **Sample/sub-sample** | | | | **Sample/sub-sample** | | | |  |
| --- | --- | --- | --- | --- | --- | --- | --- | --- | --- |
|  | **Full sample** | **Deprived sub-sample** | **Inactive sub-sample** | **Deprived / inactive sub-sample** | **Full sample** | **Deprived sub-sample** | **Inactive sub-sample** | **Deprived / inactive sub-sample** | |
| Motives n | 59,263 | 4,344 | 2,161 | 234 | 31,466 | 1,845 | 778 | 91 | |
| (Rank) Proportion of n for top 10 motives |  |  |  |  |  |  |  |  | |
| To contribute to my fitness | (1) 56.2% | (1) 52.2% | (1) 50.6% | (2) 45.3% | (1) 55.4% | (1) 53.2% | (1) 50.8% | (2) 47.3% | |
| To improve my physical health | (2) 37.0% | (2) 39.5% | (2) 49.1% | (1) 48.3% | (2) 35.0% | (2) 37.7% | (2) 45.3% | (1) 54.8% | |
| To gain a sense of personal achievement | (3) 26.9% | (3) 26.0% | (4) 25.4% | (5) 25.6% | (3) 25.5% | (3) 24.1% | (4) 23.0% | (=3) 24.7% | |
| To get a recorded time for a 5k | (4) 21.4% | (4) 22.0% | (7) 11.7% | (7) 12.8% | (4) 23.2% | (4) 23.1% | (7) 13.8% | (7) 11.8% | |
| To manage my weight | (5) 19.8% | (5) 21.4% | (3) 29.2% | (3) 32.5% | (5) 19.5% | (5) 20.4% | (3) 30.1% | (=3) 24.7% | |
| My friends, family or colleagues encouraged me to | (6) 15.2% | (7) 15.1% | (5) 24.5% | (4) 26.1% | (7) 13.3% | (8) 13.8% | (5) 20.5% | (5) 23.7% | |
| To train for another sport/event | (7) 14.2% | (8) 13.9% | (10) 6.7% | (9) 8.1% | (6) 16.0% | (7) 16.3% | (8) 9.4% | (9) *7.5%* | |
| To improve my mental health | (8) 13.0% | (6) 16.8% | (6) 17.1% | (6) 18.8% | (8) 13.2% | (6) 17.6% | (6) 18.0% | (6) 22.6% | |
| To feel part of a community | (9) 11.0% | (9) 11.3% | (9) 6.8% | (10) 6.0% | (11)10.3% | (11) 8.6% | (11) 7.1% | (10) *4.3%* | |
| To spend time outdoors | (10)10.3% | (10)10.2% | (8) 8.2% | (8) 10.3% | (9) 11.3% | (9) 12.0% | (9) 8.9% | (8) *10.8%* | |

| **(d) parkrun participation** | **Survey** | | | | **Truncated** | | | |
| --- | --- | --- | --- | --- | --- | --- | --- | --- |
|  | **Full sample** | **Deprived sub-sample** | **Inactive sub-sample** | **Deprived / inactive sub-sample** | **Full sample** | **Deprived sub-sample** | **Inactive sub-sample** | **Deprived / inactive sub-sample** |
| Years registered with parkrun n | 47,701 | 4,300 | 2,184 | 237 | 18,696 | 1,784 | 798 | 94 |
| Mean | 3.13 | 2.71 | 2.40 | 2.28 | 3.44 | 2.93 | 2.82 | 2.56 |
| SD | 2.53 | 2.30 | 1.92 | 1.80 | 2.58 | 2.25 | 1.88 | 1.83 |
| Median | 2.61 | 2.17 | 1.99 | 1.84 | 3.05 | 2.46 | 2.55 | 2.22 |
| Q1-Q3 | 0.94-4.81 | 0.72-4.20 | 0.74-3.82 | 0.68-3.46 | 1.37-5.05 | 1.07-4.36 | 1.16-4.2 | 0.90-3.70 |
| Effect size |  | 0.17 | 0.29 | 0.34 |  | 0.20 | 0.24 | 0.34 |
| Total parkruns run/walked n | 45,708 | 4,193 | 2,116 | 232 | 17,703 | 1,677 | 790 | 89 |
| Mean | 46.0 | 39.2 | 37.4 | 35.0 | 13.12 | 11.4 | 10.8 | 10.0 |
| Standard deviation | 61.1 | 54.7 | 46.9 | 48.2 | 13.91 | 12.6 | 10.4 | 10.6 |
| Median | 21 | 17 | 18 | 15 | 8 | 7 | 7 | 6 |
| Q1-Q3 | 6 - 62 | 5 - 51 | 6 - 50 | 6 – 44 | 3-18 | 3-15 | 3-15 | 2-14 |
| Effect size |  | 0.11 | 0.14 | 0.18 |  | 0.12 | 0.17 | 0.22 |
| parkruns run/walked per year n | 34,211 | 2,942 | 1,447 | 151 | 14,756 | 1,320 | 586 | 66 |
| Mean | 14.60 | 14.12 | 15.53 | 14.78 | 3.73 | 3.77 | 3.79 | 3.88 |
| Standard deviation | 12.15 | 12.02 | 12.50 | 12.67 | 2.46 | 2.50 | 2.47 | 3.25 |
| Median | 11.3 | 10.7 | 12.2 | 11.0 | 3.33 | 3.42 | 3.30 | 3.25 |
| Q1-Q3 | 4.0-23.3 | 3.9-22.5 | 4.4-25.4 | 3.9-24.1 | 1.58-5.71 | 1.50-5.90 | 1.68-5.89 | 1.89-6.57 |
| Effect size |  | 0.04 | 0.08 | 0.01 |  | 0.02 | 0.02 | 0.06 |

| **(e) physical activity at the survey** | **Survey** | | | | **Truncated** | | | |
| --- | --- | --- | --- | --- | --- | --- | --- | --- |
|  | **Full sample** | **Deprived sub-sample** | **Inactive sub-sample** | **Deprived / inactive sub-sample** | **Full sample** | **Deprived sub-sample** | **Inactive sub-sample** | **Deprived / inactive sub-sample** |
| Single activity question n | 59,967 | 4,382 | 2,183 | 236 | 31,975 | 1,869 | 790 | 95 |
| Mean | 3.59 | 3.45 | 2.41 | 2.47 | 3.57 | 3.40 | 2.35 | 2.52 |
| Standard deviation | 1.77 | 1.81 | 1.67 | 1.71 | 1.81 | 1.88 | 1.74 | 1.87 |
| Median | 3 | 3 | 2 | 2 | 3 | 3 | 2 | 2 |
| Q1 – Q3 | 2 – 5 | 2 – 5 | 1 – 3 | 1 - 3 | 2 – 5 | 2 – 5 | 1 – 3 | 1 – 4 |
| Effect size |  | 0.08 | 0.67 | 0.64 |  | 0.09 | 0.67 | 0.58 |
| IPAQ n | 45,496 | 3,303 | 1,568 | 171 | 23,250 | 1,380 | 545 | 67 |
| Proportion low or moderate physical activity | 35.8% | 38.0% | 62.2% | 59.6% | 35.9% | 38.9% | 62.8% | 56.7% |
| Proportion high physical activity (health enhancing) | 64.2% | 62.0% | 37.8% | 40.4% | 64.1% | 61.1% | 37.2% | 43.3% |
