# Supplementary material for: Exploring the benefits of participation in community-based running and walking events: a cross-sectional survey of parkrun participants
Source: BMC Public Health. 2021 Nov 2;21:1978. doi: 10.1186/s12889-021-11986-0 (PMC8561845; doi:10.1186/s12889-021-11986-0)
Supplement: Supplementary file 4 — Additional file 4 Perceived impact with truncated sample. Perceived impact of running or walking at parkrun using the question “Thinking about the impact of parkrun on your health and wellbeing, to what extent has running or walking at parkrun changed”. Allowed responses were ‘much worse, worse, no impact, better, much better’. Data in the table is a combined value for ‘better’ and ‘much better’. Results are compared to a truncated sample who participated in ≤8.85 parkruns per year. [file 12889_2021_11986_MOESM4_ESM.docx]

**Additional File 4**

Perceived impact of running or walking at parkrun using the question “Thinking about the impact of parkrun on your health and wellbeing, to what extent has running or walking at parkrun changed”. Allowed responses were ‘much worse, worse, no impact, better, much better’. Data in the table is a combined value for ‘better’ and ‘much better’.

| **Reporting 'better' or 'much better'** | **Survey** | | | | **Truncated sample** | | | |
| --- | --- | --- | --- | --- | --- | --- | --- | --- |
| **Reporting 'better' or 'much better'** | **Full sample** | **Deprived** | **Inactive** | **Deprived / inactive** | **Full sample** | **Deprived** | **Inactive** | **Deprived / inactive** |
| Your sense of personal achievement n | 56,276 | 4,131 | 2,071 | 223 | 29,382 | 1,742 | 732 | 85 |
| % | 90.7% | 91.7% | 93.4% | 93.3% | 87.4% | 88.2% | 89.1% | 91.8% |
| Fitness n | 56,269 | 4,125 | 2,072 | 223 | 29,378 | 1,740 | 735 | 86 |
| % | 89.3% | 91.3% | 92.9% | 92.4% | 85.2% | 86.3% | 86.1% | 84.9% |
| Physical health n | 56,262 | 4,134 | 2,077 | 225 | 29,360 | 1,743 | 735 | 87 |
| % | 84.7% | 87.0% | 88.5% | 89.8% | 80.3% | 81.2% | 81.8% | 83.9% |
| Happiness n | 56,217 | 4,126 | 2,068 | 224 | 29,342 | 1,740 | 730 | 86 |
| % | 78.8% | 81.8% | 80.8% | 83.5% | 73.8% | 74.4% | 74.1% | 77.9% |
| The amount of time you spend outdoors n | 56,251 | 4,134 | 2,076 | 225 | 29,358 | 1,743 | 735 | 87 |
| % | 74.1% | 78.7% | 82.1% | 85.8% | 68.6% | 71.8% | 73.7% | 79.3% |
| Your enjoyment of competing n | 56,253 | 4,126 | 2,072 | 224 | 29,369 | 1,739 | 733 | 86 |
| % | 72.7% | 74.2% | 70.6% | 70.1% | 68.2% | 68.5% | 62.5% | 68.6% |
| How much you feel part of a community n | 56,217 | 4,120 | 2,076 | 225 | 29,346 | 1,738 | 734 | 87 |
| % | 69.7% | 70.6% | 68.2% | 69.8% | 62.5% | 61.6% | 57.1% | 63.2% |
| Mental health n | 56,215 | 4,127 | 2,074 | 225 | 29,342 | 1,743 | 735 | 87 |
| % | 69.3% | 73.9% | 72.3% | 76.4% | 66.1% | 68.8% | 68.6% | 72.4% |
| Confidence n | 56,225 | 4,132 | 2,075 | 225 | 29,340 | 1,742 | 735 | 87 |
| % | 61.3% | 66.3% | 64.0% | 70.7% | 57.8% | 61.4% | 58.9% | 67.8% |
| Your ability to be active in a safe environment n | 56,193 | 4,122 | 2,072 | 225 | 29,328 | 1,739 | 733 | 87 |
| % | 59.9% | 65.3% | 69.3% | 72.4% | 56.4% | 59.7% | 61.9% | 65.5% |
| Number of new people you meet n | 56,237 | 4,127 | 2,075 | 225 | 29,351 | 1,739 | 735 | 87 |
| % | 57.5% | 58.7% | 55.8% | 60.9% | 47.2% | 45.5% | 39.7% | 50.6% |
| Ability to control your weight n | 56,208 | 4,124 | 2,074 | 224 | 29,336 | 1,740 | 733 | 86 |
| % | 52.3% | 54.7% | 56.3% | 54.0% | 47.8% | 47.9% | 49.5% | 45.3% |
| Overall lifestyle choices (e.g. diet & smoking) n | 56,209 | 4,118 | 2,074 | 224 | 29,332 | 1,736 | 733 | 86 |
| % | 51.8% | 56.4% | 57.2% | 65.2% | 47.7% | 49.8% | 49.4% | 57.0% |
| The amount of time you spend with friends n | 56,181 | 4,125 | 2,073 | 224 | 29,320 | 1,741 | 733 | 87 |
| % | 41.1% | 42.4% | 41.1% | 46.0% | 34.8% | 34.3% | 33.7% | 40.2% |
| Amount of time you spend with family n | 56,140 | 4,123 | 2,071 | 224 | 29,296 | 1,735 | 735 | 86 |
| % | 27.7% | 26.2% | 31.7% | 29.5% | 25.2% | 22.0% | 29.8% | 33.7% |
